# What makes *Xanthomonas albilineans* unique amongst xanthomonads?

**DOI:** 10.3389/fpls.2015.00289

**Published:** 2015-04-24

**Authors:** Isabelle Pieretti, Alexander Pesic, Daniel Petras, Monique Royer, Roderich D. Süssmuth, Stéphane Cociancich

**Affiliations:** ^1^UMR BGPI, Cirad, Montpellier, France; ^2^Institut für Chemie, Technische Universität Berlin, Berlin, Germany

**Keywords:** *Xanthomonas albilineans*, leaf scald disease of sugarcane, genomic features, albicidin, NRPS and PKS genes

## Abstract

*Xanthomonas albilineans* causes leaf scald, a lethal disease of sugarcane. Compared to other species of *Xanthomonas, X. albilineans* exhibits distinctive pathogenic mechanisms, ecology and taxonomy. Its genome, which has experienced significant erosion, has unique genomic features. It lacks two loci required for pathogenicity in other plant pathogenic species of *Xanthomonas*: the xanthan gum biosynthesis and the Hrp-T3SS (hypersensitive response and pathogenicity-type three secretion system) gene clusters. Instead, *X. albilineans* harbors in its genome an SPI-1 (*Salmonella* pathogenicity island-1) T3SS gene cluster usually found in animal pathogens. *X. albilineans* produces a potent DNA gyrase inhibitor called albicidin, which blocks chloroplast differentiation, resulting in the characteristic white foliar stripe symptoms. The antibacterial activity of albicidin also confers on *X. albilineans* a competitive advantage against rival bacteria during sugarcane colonization. Recent chemical studies have uncovered the unique structure of albicidin and allowed us to partially elucidate its fascinating biosynthesis apparatus, which involves an enigmatic hybrid PKS/NRPS (polyketide synthase/non-ribosomal peptide synthetase) machinery.

## Introduction

*Xanthomonas albilineans* (Ashby) Dowson is known to invade the xylem of sugarcane and to cause leaf scald disease (Rott and Davis, 2000; Birch, 2001). Symptoms of this disease vary from a single, white, narrow, sharply defined stripe to complete wilting and necrosis of infected leaves, leading to plant death. Dissemination of *X. albilineans* occurs mainly mechanically through use of contaminated harvesting tools and by distribution and planting of infected cuttings. However, aerial transmission and potential for epiphytic survival have also been reported for this pathogen (Autrey et al., 1995; Daugrois et al., 2003; Champoiseau et al., 2009).

*Xanthomonas albilineans* is a representative of the genus *Xanthomonas*, members of which are exclusively Gram-negative plant-associated bacteria that collectively cause dramatic damage to hundreds of plant species of ornamental or agronomical interest. Indeed, both monocotyledonous (e.g., rice, sugarcane, or banana) and dicotyledonous (e.g., citrus, cauliflower, bean, pepper, cabbage, and tomato) plants are targeted worldwide by various *Xanthomonas* species. While sharing numerous phenotypic characteristics, at least 27 species and over 120 pathovars (variants of pathogeny) of the genus *Xanthomonas* are currently recognized. Each pathovar individually exhibits a very restricted host range and/or tissue-specificity and this leads to clustering of bacterial strains causing similar symptoms on the same host.

Multilocus sequence analysis (MLSA) with four housekeeping genes resulted in the distribution of *Xanthomonas* species in two clades. The main one contains the majority of species whereas the secondary clade contains *X. albilineans*, *Xanthomonas sacchari*, *Xanthomonas theicola*, *Xanthomonas hyacinthi*, and *Xanthomonas translucens* (Young et al., 2008). Phylogenetic analyses with the *gyrB* sequence indicate that this secondary group also contains several uncharacterized species of *Xanthomonas* isolated mainly on rice, banana or sugarcane (Studholme et al., 2011, 2012). Intriguingly, two multiMLSA studies with 28 genes and 228 genes, respectively, in which *X. albilineans* is the only representative of this secondary clade, resulted in the branching of *Xylella fastidiosa* between *X. albilineans* and the main clade (Rodriguez-R et al., 2012; Naushad and Gupta, 2013). *X. fastidiosa* is a xylem-limited bacterium which is insect-vectored to a variety of diverse hosts, has a reduced genome and lacks the Hrp-T3SS (hypersensitive response and pathogenicity–type III secretion system; Simpson et al., 2000).

Analysis of the *X. albilineans* genome has revealed unusual features compared to other xanthomonads, the most prominent being the absence of the Hrp-T3SS gene cluster and the occurrence of genome erosion. Furthermore, to our knowledge, *X. albilineans* is the only xanthomonad that produces the phytotoxin albicidin. This mini-review aims to summarize the characteristics that, taken together, make *X. albilineans* so unique.

## Genome Erosion

The genome of *X. albilineans* strain GPE PC73 has been fully sequenced and annotated. It consists of a 3,768,695-bp circular chromosome with a G+C content of 63%, and three plasmids of 31,555-bp, 27,212-bp and 24,837-bp, respectively (Pieretti et al., 2009). This genome size is much smaller than that of any other xanthomonad sequenced to date (commonly ∼5 Mb). Examination of the genome of strain GPE PC73 together with OrthoMCL comparative analyses performed with other sequenced xanthomonads highlights several genomic features that distinguish *X. albilineans* from its near relatives (Pieretti et al., 2009, 2012; Marguerettaz et al., 2011; Royer et al., 2013).

Orthologous analyses show that *X. albilineans* and *X. fastidiosa* have experienced a convergent genome reduction during their respective speciation, with a more extensive genome reduction for *X. fastidiosa* (Pieretti et al., 2009). Based on these analyses, *X. albilineans* has lost at least 592 genes that were present in the last common ancestor of the xanthomonads. Interestingly, most of these ancestral genes are conserved in the genome of *X. sacchari* strains NCPPB4393 and LMG 476 and *Xanthomonas* spp. strains NCPPB1131 and NCPPB1132, which are the sequenced strains phylogenetically closest to *X. albilineans* (Studholme et al., 2011, 2012; Pieretti et al., 2015). This indicates that genome erosion is specific to *X. albilineans*. Convergent genome erosion of *X. albilineans* and *X. fastidiosa* could be linked to a similar adaptation to a xylem-invading lifestyle in which interactions with living plant tissues are minimal (Pieretti et al., 2009). More recently, a study of the somewhat reduced genome of *Xanthomonas fragariae* (4.2 Mb) led to the hypothesis that the convergent genome reduction observed in some xanthomonads could be linked to their endophytic lifestyle and typically to their commitment to a single host (Vandroemme et al., 2013).

Compared to other xanthomonads, a low number of insertion sequences (IS) has been found in the genome of *X. albilineans*. Taken together with a limited recombination of the chromosome and a GC skew pattern containing a low number of distortions, it was postulated that genome erosion of *X. albilineans* was mainly not due to IS and other mechanisms were proposed for this erosion (Pieretti et al., 2009). The low number of IS could be linked to the activity of CRISPR (clustered regularly interspaced short palindromic repeats) systems. Strain GPE PC73 of *X. albilineans* possesses two CRISPR loci. The first one, CRISPR-1, is conserved in *X. oryzae* pv. *oryzae*, *X. axonopodis* pv. *citri*, *X. campestris* pv. *vasculorum*, and *X. campestris* pv. *musacearum*. The second, CRISPR-2, is present in *X. campestris* pv. *raphani* (Pieretti et al., 2012). Interestingly, many spacers of CRISPR-1 and CRISPR-2 of strain GPE PC73 are identical to IS or phage-related DNA sequences present on the chromosome of this strain (Pieretti et al., 2012).

## Specific Genes Linked to a Xylem-Invading Lifestyle

Although determinants for host- or tissue-specificity of *X. albilineans* remain unclear, the presence in its genome of genes encoding cell-wall-degrading enzymes (CWDEs) with specific features is probably important for its ability to spread in xylem and for pathogenicity. Indeed, all CWDEs from *X. albilineans* harbor a cellulose-binding domain (CBD) and a long linker region both adapted to the utilization of cell-wall breakdown products as carbon source and to the ability to spread in sugarcane xylem vessels (Pieretti et al., 2012). These enzymes may also be required to disrupt pit membranes in sugarcane, thereby promoting propagation of the bacteria in the plant. Interestingly, *X. fastidiosa* also encodes two CWDEs containing a long linker and a CBD. It has been shown that one of these two CWDEs is involved in the spread of *X. fastidiosa* in the xylem by increasing the pore size of pit membranes. CWDEs are therefore considered as virulence factors (Roper et al., 2007; Chatterjee et al., 2008; Pérez-Donoso et al., 2010). TonB-dependent transporters (TBDTs) may be used by *X. albilineans* to transport cell-wall-degrading products resulting from the activity of CWDEs, and thus may facilitate spread of the organism in the nutrient-poor conditions prevailing in the xylem of sugarcane. In the genome of *X. albilineans*, 35 TBDT genes have been identified, including one specific to this species and two others that are functionally associated to pathogenicity of the bacterium (Rott et al., 2011; Pieretti et al., 2012).

## Lack of Hrp-T3SS

Most phytopathogenic bacteria rely on the type III secretion system (T3SS) of the hypersensitive response and pathogenicity family (Hrp1 and Hrp2, respectively). This syringe-like apparatus allows pathogens to deliver, into their host cells, proteins (type III effectors) that modulate plant physiology and immunity for the benefit of the pathogen. Interestingly, genes encoding the injectisome and associated effectors of the Hrp-T3SS are missing in the genome of *X. albilineans*, as is also the case in the genomes of *X. sacchari* strains NCPPB4393 and LMG 476 and *Xanthomonas* spp. strains NCPPB1131 and NCPPB1132 (Studholme et al., 2011, 2012; Pieretti et al., 2015). Yet, an Hrp system is present in other close neighbor species of *X. albilineans*, such as *X. translucens* pv. *graminis* strain 29, *X. translucens* pv. *translucens* strain DSM18974, and *X. translucens* strain DAR 61454 (Wichmann et al., 2013; Gardiner et al., 2014). Although the Hrp-T3SS is described as a crucial key component in plant–host interactions for most *Xanthomonas* spp, it seems not to be essential in *X. translucens* pv. *graminis* strain 29 for xylem colonization, even though it is involved in symptom development (Ryan et al., 2011; Wichmann et al., 2013). Similarly, despite being devoid of any Hrp T3SS, *X. albilineans* displays pathogenicity and is able to cause serious damage to sugarcane.

## Acquisition of a SPI-1 T3SS

The annotated sequence of the genome of *X. albilineans* strain GPE PC73 reveals the presence of a T3SS belonging to the *Salmonella* pathogenicity island-1 (SPI-1) injectisome family. Genes encoding this system are located near the terminus of the replication site of the chromosome and were probably acquired by lateral gene transfer. This secretion system, found mainly in mammals and insects bacterial pathogens or symbionts, exhibits high similarity to that described in *Burkholderia pseudomallei*—a human pathogen causing melioidosis (Stevens et al., 2002). The SPI-1 needle-like assemblies of *X. albilineans* strain GPE PC73 and *B. pseudomallei* strain K96243 are homologous. Both species share all but two genes—*orgA* and *orgB*, encoding putative oxygen-regulated invasion proteins involved in type three secretion that are not conserved in *B. pseudomallei*. The genome composition of the SPI-1 T3SS in *X. albilineans* additionally includes genes encoding translocon components (*xipB*, *xipC*, and *xipD*), injectisome components (*xsaJ* to *xsaS* and *xsaV* to *xsaZ*) and a chaperone (*xicA*). Furthermore, the locus contains 15 additional genes referred to as *xapA*–*xapO*, encoding hypothetical proteins. These genes, which show homology neither to sequences from *B. pseudomallei* nor to sequences available from protein sequence databases, are specific to *X. albilineans* and their products represent good candidates to be considered as effectors for this SPI-1 T3SS (Marguerettaz et al., 2011). Interestingly, this SPI-1 T3SS is conserved in *Xanthomonas axonopodis* pv. *phaseoli* strains CFBP 2534, CFBP 6164 and CFBP 6982, which moreover possess a second T3SS belonging to the Hrp2 family (Alavi et al., 2008; Marguerettaz et al., 2011). Pathogenicity of *X. albilineans* strains seems not to be linked to the presence of the SPI-1 T3SS in their genome; besides, no SPI-1 T3SS locus has been identified in strain PNG130 of *X. albilineans* even though it is able to spread in sugarcane. Functional analyses showed that, *in planta*, multiplication of a SPI-1 T3SS knockout mutant of *X. albilineans* was not impaired when compared to the wild-type, indicating that the SPI-1 T3SS is not required for spread in sugarcane vessels or for development of leaf scald symptoms. The role of the SPI-1 T3SS of *X. albilineans* remains unclear, although it has been conserved during its evolution in *X. albilineans* without frame-shifting indels or nonsense mutations (Marguerettaz et al., 2011). It remains possible, in conditions other than those tested with our knockout mutant, that the SPI-1 T3SS system may be required for interaction with sugarcane, as in the case of SPI-1 of *Salmonella*, which is involved in interactions with *Arabidopsis thaliana* (Schikora et al., 2011). The SPI-1 T3SS system may also be associated with other aspects of the *X. albilineans* lifestyle, e.g., an involvement in adherence as reported for *Erwinia tasmaniensis* (Kube et al., 2008) or in formation of pellicle or biofilm-like structures (Jennings et al., 2012), which could be related to epiphytic survival on sugarcane leaves. Although no insect vector has been identified for *X. albilineans* to date, we cannot rule out that the SPI-1 T3SS could be involved in insect association or might mediate persistence of the bacterium in an insect vector as was shown for *Pantoea stewartii* (Correa et al., 2012).

## Lack of T6SS and the Xanthan Gum Gene Cluster

*Xanthomonas albilineans* lacks two other major pathogenicity factors that are common features of most xanthomonads. First, it lacks the gum gene cluster for extracellular polysaccharide (EPS) synthesis. This gene cluster is responsible for biofilm and xanthan gum formation, and is associated with pathogenesis in xanthomonads (Katzen et al., 1998; Kim et al., 2009; Galván et al., 2012). Exceptions are *X. fragariae*, which lacks the *gumN*, *gumO* and *gumP* genes, and *X. albilineans*, which lacks the complete set of gum genes, indicating those are not essential for virulence of both these pathogens (Pieretti et al., 2012; Vandroemme et al., 2013).

*Xanthomonas albilineans* is also devoid of any type VI secretion system (T6SS) described in other xanthomonads, as for example in *Xanthomonas fuscans* pv. *fuscans* strain 4834-R and *Xanthomonas citri* subsp. *citri* strain 306, which each contain a single T6SS (Potnis et al., 2011; Darrasse et al., 2013) or *X. translucens* strain DAR61454, which encodes two distinct T6SS (Gardiner et al., 2014). Structurally, the T6SS looks like an inverted bacteriophage. Functionally, this system is able to interact with both eukaryotic and prokaryotic cells by delivering effectors or toxins into host cells to subvert the signaling process to its own advantage, but also into other bacteria from the same habitat to outcompete them during infection (Filloux, 2013; Russell et al., 2014). Despite its multifunctional roles during host–pathogen interactions, the lack of T6SS in *Xanthomonas campestris* pv. *campestris* strain 8004, *Xanthomonas gardneri* strain 101, and *X. albilineans* seems to have no effect on pathogenesis of these xanthomonads.

## Albicidin and Other Non-Ribosomally Synthesized Peptides

**FIGURE 1:**
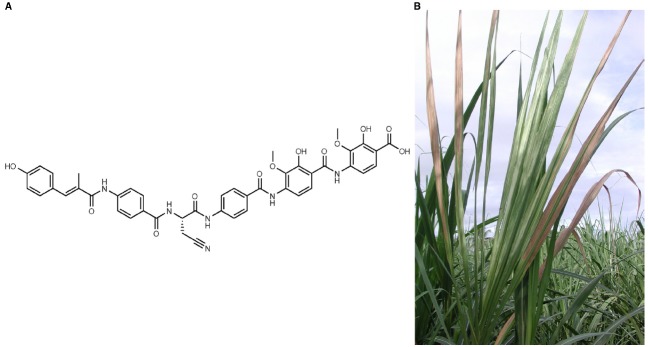
***Xanthomonas albilineans* produces the phytotoxin albicidin—a potent gyrase inhibitor that blocks chloroplast differentiation, resulting in sugarcane leaf scald disease symptoms. (A)** Structure of albicidin, a hybrid PKS/NRPS compound with unique composition including *p*-aminobenzoic acid and cyanoalanine. **(B)** Diseased sugarcane plant with characteristic leaf scald symptoms: white foliar bleaching and necrosis of infected leaves (© J. H. Daugrois/Cirad).

A unique feature of *X. albilineans* is the production of albicidin—a phytotoxin causing the white foliar stripe symptoms characteristic of leaf scald disease of sugarcane (Birch and Patil, 1985). Albicidin is a potent DNA gyrase inhibitor that blocks the differentiation of chloroplasts (Figure 1). It also targets bacterial gyrase by a mechanism different from that of other DNA gyrase inhibitors like coumarins and quinolones (Hashimi et al., 2007). This mode of action accounts for the potent antibacterial activity of albicidin, which inhibits the growth of Gram-positive and Gram-negative pathogenic bacteria at nanomolar concentrations (Birch and Patil, 1985). Albicidin gives a competitive advantage to *X. albilineans* against other bacteria within the xylem vessels of sugarcane (Magnani et al., 2013). Interestingly, two sugarcane-living bacteria harbor an albicidin resistance gene: *Leifsonia xyli* (Monteiro-Vitorello et al., 2004) and *Pantoea dispersa* (Zhang and Birch, 1997).

Albicidin is produced by a hybrid polyketide synthase (PKS)/non-ribosomal peptide synthetase (NRPS) enzyme complex. PKS and NRPS genes are often clustered together with a large set of regulatory, transport or modification (tailoring) genes, as well as genes involved in the biosynthesis of non-proteinogenic amino acids. In addition to a phosphopantetheinyl transferase required for activation of the PKS/NRPS system and a HtpG chaperone, the role of which remains unclear, a locus (*alb* cluster) containing 20 genes is required for albicidin biosynthesis. Among these 20 genes, 3 encode the PKS/NRPS system; 15 others act as transport, regulatory, modification or resistance genes (Royer et al., 2004).

Non-ribosomal peptide synthetases are multimodular megasynthetases used by bacteria and fungi to produce peptides in a ribosome-independent manner (Strieker et al., 2010). Each module governs the specific incorporation of an amino acid substrate based on signature sequences in the adenylation (A) domains (Stachelhaus and Marahiel, 1995), which are loaded onto peptidyl carrier protein (PCP) domains. Elongation of the peptide is mediated by condensation (C) domains present within each module. PKSs function according to the principles of fatty acid biosynthesis (Weissman and Leadlay, 2005).

For decades, the structure elucidation of albicidin was impeded by its extremely low production yield by *X. albilineans*. A first step to overcome this bottleneck was achieved by transferring the biosynthetic genes into a heterologous host, namely *X. axonopodis* pv. *vesicatoria*, resulting in a significant increase in albicidin production (Vivien et al., 2007). Extensive HPLC purification of albicidin and thorough analysis of the purified compound by means of mass spectrometry and nuclear magnetic resonance spectroscopy then allowed us to unravel its unique structure (Figure 1). Albicidin proved to be a linear pentapeptide composed of cyanoalanine and *p*-amino benzoic acids N-terminally linked to a *p*-coumaric acid derivative (Cociancich et al., 2015). Although over 500 different monomers (amino acid substrates) have been identified to date as being incorporated by NRPS systems, elucidation of the structure of albicidin revealed for the first time the incorporation by NRPSs of cyanoalanine and *p*-amino benzoic acids. Moreover, the incorporation of *p*-amino benzoic acids is the first example of incorporation of a δ-aminoacid by NRPSs, since all NRPSs described to date incorporate only α or β aminoacids. The use of unusual amino acid substrates is linked to unique features that were identified *in silico* 10 years ago within the albicidin NRPS modules sequence (Royer et al., 2004). The formation and incorporation of cyanoalanine most likely occurs *in situ* through an additional module present in the PKS-NRPS assembly line that was investigated in one of our present studies (Cociancich et al., 2015).

Chemical synthesis of albicidin is now available, allowing both production of high quantities of the compound for further study of its mode of action and activity spectrum, and the synthesis of analogs (Kretz et al., 2015). The uniqueness of its structure and the specific mode of action of this compound make albicidin a strong lead structure for antibiotic development.

Data mining of the genome of *X. albilineans* strain GPE PC73 has led to the identification, in addition to the albicidin biosynthesis locus, of five other NRPS loci (Pieretti et al., 2012; Royer et al., 2013). The first, named Meta-B, encodes megasynthases performing peptidic elongation of a 16-amino acid lipopeptide. This locus also encodes a transcription regulator belonging to the AraC family, a cyclic peptide transporter, and enzymes involved in biosynthesis of the non-proteinogenic amino acids di-amino butyric acid and dihydroxyphenylglycine. Interestingly, the NRPS locus Meta-B has been identified in the genome of strains of three other *Xanthomonas* species, namely *Xanthomonas oryzae* pv. *oryzae* strains BAI3 and X11-5A, *X. translucens* strain DAR61454 and *Xanthomonas* spp. strain XaS3 (Royer et al., 2013). Despite a similar organization of the genes within these loci, the *in silico* prediction of the sequences of the peptides produced indicates that each strain produces a different lipopeptide.

Two other NRPS gene clusters, Meta-A and Meta-C, have been identified in the genome of *X. albilineans* strain GPE PC73. They encode megasynthases that perform the biosynthesis of peptides of 12 and 7 amino acids, respectively. A partial sequence has been predicted for each of these peptides (Royer et al., 2013).

Finally, two short NRPS genes have also been identified on the chromosome of *X. albilineans*: they both encode only one NRPS module. Interestingly, there is an overlap between both these genes and a gene encoding a glycosyltransferase. It has been hypothesized that these genes encode glycosylated amino acids, to which, however, no precise function could yet be attributed (Royer et al., 2013).

## Conclusion

Although most xanthomonads require pathogenicity factors such as *gum* genes, T3SS Hrp and T6SS for survival, growth and spread within host plants, *X. albilineans* lacks these pathogenicity factors, *de facto* reducing its artillery to circumvent sugarcane defense mechanisms and innate immunity. While being “disarmed” could be disadvantageous for a vascular plant pathogen, *X. albilineans* remains able to invade and spread in sugarcane, suggesting that it uses other strategies, such as stealth, i.e., being unobtrusive *in planta*, to minimize inducible host defense responses. On the other hand, the reduced genome of *X. albilineans* has specific features that may be involved in the adaptation of the bacterium to live and spread in sugarcane xylem vessels. For example, specific CWDEs and TBDTs appear to be optimized for life in the nutrient-poor sugarcane xylem environment. The uniqueness of *X. albilineans* resides also in the production of the phytotoxin and antibiotic albicidin. The recently unraveled structure and concomitant development of a chemical synthesis protocol for this compound leads to additional prospects for its use in the antibiotherapy field. According to the specificities deriving from the biological, biochemical, phylogenetic and genomic analyses described in this review, one can truly say that *X. albilineans* is quite unique amongst the genus *Xanthomonas*.

### Conflict of Interest Statement

The authors declare that the research was conducted in the absence of any commercial or financial relationships that could be construed as a potential conflict of interest.
